# On the ridge of instability in ferrofluidic Couette flow via alternating magnetic field

**DOI:** 10.1038/s41598-021-84175-z

**Published:** 2021-02-25

**Authors:** Sebastian Altmeyer

**Affiliations:** Castelldefels School of Telecom and Aerospace Engineering, Universitat Politècnica de Catalunya, 08034 Barcelona, Spain

**Keywords:** Fluid dynamics, Applied mathematics, Nonlinear phenomena, Statistical physics, Computational science

## Abstract

There is a huge number of natural and industrial flows, which are subjected to time-dependent boundary conditions. The flow of a magnetic fluid under the influence of temporal modulations is such an example. Here, we perform numerical simulations of ferrofluidic Couette flow subject to time-periodic modulation (with frequency $\Omega _H$) in a spatially homogeneous magnetic field and report how such a modulation can lead to a significant Reynolds number *Re* enhancement. Consider a modified Niklas approximation we explain the relation between modulation amplitude, driving frequency and stabilization effect. From this, we describe the system response around the primary instability to be sensitive/critical by an alternating field. We detected that such an alternating field provides an *easy* and in particular *accurate* controllable key parameter to trigger the system to change from subcritical to supercritical and vice versa. Our findings provide a framework to study other types of magnetic flows driven by time-dependent forcing.

## Introduction

The spontaneous formation of spatial and temporal patterns can be observed in many physical, chemical, and biological systems that are driven out of the equilibrium^1^. A well known and extensively investigated hydrodynamic pattern forming system is the Taylor–Couette system (TCS)^2,3^ consisting of two concentric cylinders with different radii which can rotate independently of each other. Typical control parameter is the dimensionless Reynolds numbers *Re*, pondering the effects of inertia and viscosity.

The effect of time-periodic forcing in TCS has been investigated in numerous works^4–11^. Such a forcing can be realized by (axial or azimuthal) oscillation of one or both cylinders, further by pulsation of axial imposed flow or radial through flow, with the latter requiring porous cylinder walls. Considering magnetic fluids, e.g. *ferrofluids*^12^, offers an alternative method to realize such a periodic forcing.

Ferrofluids are manufactured fluids, which consist of dispersions of magnetized nanoparticles in a variety of liquid carriers^12,13^. In order to avoid or at least to minimize agglomeration effects they are stabilized by the addition of a surfactant monolayer surrounding the particles. If no magnetic field is present the fluid behaves as a classical fluid with zero net magnetization as the magnetic nanoparticles are randomly orientated. In this scenario the fluid’s viscosity and density experience typically very small alteration due to the presence of the nanoparticles itselves. The latter however, significantly changes when a magnetic field is applied. For a sufficiently strong magnetic field, the ferrofluid flows toward regions of the magnetic field. This coincide with a change in the fluid’s properties such as the viscosity. This, also known as the magneto-viscous effect^14,15^, can *significantly* change the hydrodynamics of the system. Applications using ferrofluids are versatile and can be found in different fields and areas, spanning from separation over mechanical positioning towards medical applications^16,17^.

Magnetic fluids such as ferrofluids show a strong paramagnetic behavior if exposed to an external magnetic field^12,18^. To date, most numerical and experimental studies of ferrofluidic flows in TCS consider static magnetic fields have been conducted considering different field orientations, internal magnetization, agglomeration and other effects^15,18–28^. All these works came to one *common* conclusion that the basic state (Circular Couette flow, CCF) in the system becomes *stabilized* with increasing magnetic field strength. Thus the thresholds for the first appearing instability (here a centrifugal instability^2^) is shifted to larger *Re*. For here considered TCS with outer cylinder at rest, the homogeneous CCF state grows into the structured solution of Taylor vortex flow (TVF) crossing the critical threshold and breaking the axial translation invariance.

One possibility to introduce periodic forcing into the system is the realization by periodic modulation of the external magnetic field, which results in a time-dependent magnetic parameter. In the current paper this is the time-dependent Niklas parameter^19^.

To date, studies of ferrofluid under alternating magnetic fields are relatively rare and if conducted special attention has been given to their heat behavior^29,30^. Most prominent observation for ferrofluidic flows under alternating fields is the fact that sufficiently high modulation frequency field will force a faster rotation of the particles. This spins up the fluid and thus reduces it’s viscosity while in contrast a static field hindrance the free rotation of the magnetic material resulting in an increase of viscosity. In literature occasionally referred to as *negative viscosity* of the ferrofluid^20,31^. Other works also focussed on the dependence of particle agglomeration under rotating field^32^.

Knowing about the stabilization effect of an applied magnetic field, the question arises how does the system response under an alternating magnetic field for control parameters around the bifurcation threshold of the first instability. To understand the dynamics and system response while “walking” along this ridge of instability between sub- and supercritical states is a main focus of the present work.

Here we numerically study modulated ferrofluidic Couette flow within a wide range modulation frequency and amplitude and observe a *significant enhancement* in system stability (in $Re \approx 220\%$). Further we demonstrate that such an alternating magnetic field provides an *easy controllable* and quite *accurate* way to balance the system and walk along the narrow ridge of instability. As such it allows to drive the system to be subcritical or supercritical.

## Results

### System parameters

**Figure 1:**
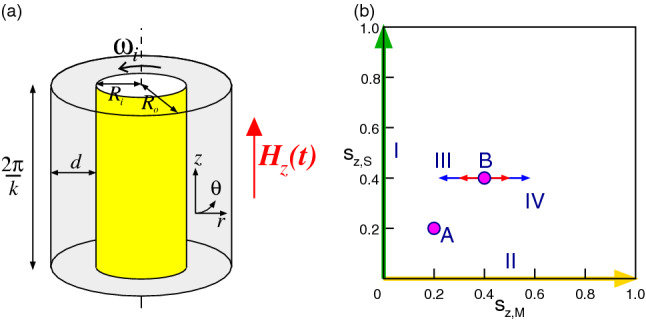
System and explored parameter space. (**a**) Schematic of the Taylor-Couette system (TCS) with an external applied homogeneous magnetic field $\mathbf{H}_z(t) = [H_S + H_M \sin {(\Omega _H t)}] \mathbf{e}_z$. (**b**) The arrows I and II indicate the investigated parameter space spanned by $s_{z,S}\in [0,1]$ and $s_{z,M}\in [0,1]$. Point A gives the parameters for supercritical flow at $Re=100$. III and IV correspond to the set of parameters around the onset of stability in point B $Re=80$.

In TCS (Fig. 1a) the flow strength is represented in terms of the Reynolds number $Re=\omega _i r_i d/ \nu$ (the ratio between inertia and viscous forces), which is a very well suited parameter to describe the driving of the system^33^. Here $r_i$ is the non-dimensionalized radius and $\omega _i$ the angular velocity of the inner cylinder. No-slip boundary conditions are used on the cylinder surfaces. The system can be characterized in the cylindrical coordinate system $(r,\theta ,z)$ by the velocity field $\mathbf{u}=(u,v,w)$ and the corresponding vorticity field $\nabla \times \mathbf{u} = (\xi ,\eta ,\zeta )$. The radius ratio of the cylinders, is kept fixed at 0.5. The time, and length scales are made dimensionless by diffusion time $d^2/\nu$ and gap width *d*. The pressure in the fluid is normalized by $\rho \nu ^2/d^2$.

In the periodically modulated TCS, we give a sinusoidal modulation signal to the external magnetic field (parallel to the system symmetry (*z*) axis, uniform in space and harmonic in time) as $\mathbf{H}_z = [H_S + H_M \sin {(\Omega _H t)}] \mathbf{e}_z$. As earlier reported such a pure axial magnetic field does not change the system symmetry and only shift the stability thresholds^23,24^. The magnetic field $\mathbf{H}$ and the magnetization $\mathbf{M}$ are conveniently normalized by the quantity $\sqrt{\rho /\mu _0} \nu /d$, with free space permeability $\mu _0$. By using a modified Niklas approach^19,24^ the effect of the magnetic field and the magnetic properties of the ferrofluid on the velocity field can be characterized by the (time dependent) Niklas function (see “Methods” section for details)

1 $$\begin{aligned} s_z(t) = s_{z,S} + s_{z,M} \sin {(\Omega _H t)}, \end{aligned}$$

with three control parameters, $s_{z,S}$ being the *static contribution* of the driving, $s_{z,M}$ the *modulation amplitude*, and $\Omega _H$ the *modulation frequency*. See “Methods” section for more details.

### Explored parameter space

We explore the parameter space within $s_{z,S}\in [0,1]$ and $s_{z,M}\in [0,1]$. The trajectories I and II shown in the parameter space of Fig. 1b represent pure static and pure alternating magnetic fields, respectively. Point A presents the parameters for supercritical flow (Taylor Vortex flow, TVF) at $Re=100$. The trajectories III and IV highlight the parameters at which we provide a more detailed study around the onset of instability at point B for $Re=80$ (cf. Fig. 5).

### Stability behavior

#### Static magnetic fields ($s_{z,M}=0$)

**Figure 2:**
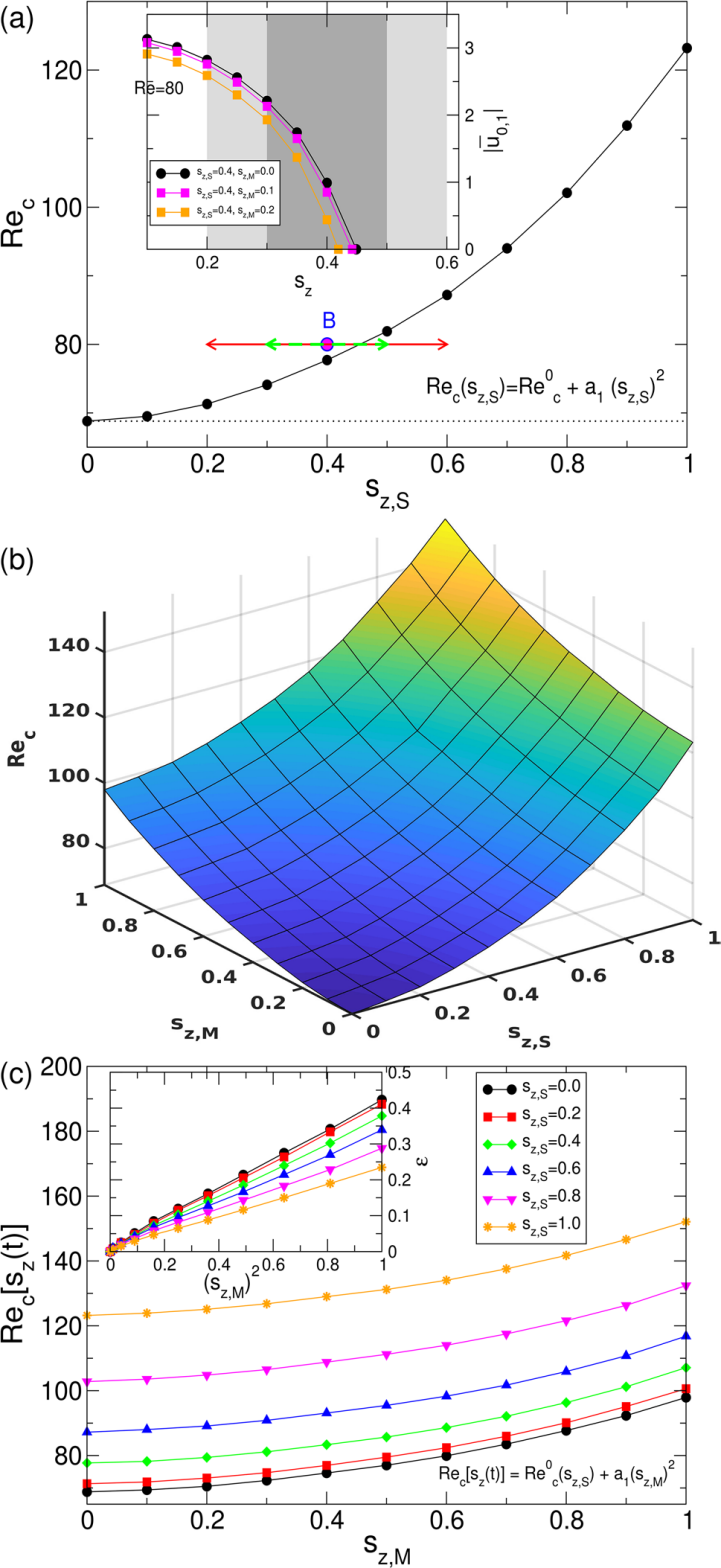
Stability in magnetic fields. (**a**) Stability boundaries for a static magnetic field as a function of $s_{z,S}$. The curve can be approximated by the function $Re_{c}(s_{z,S}) = Re^0_{c} + a_1 s_{z,S}^2$ ($a_1=53.3$), where $Re^0_{c}$ is the stability threshold in absence of any magnetic field. Horizontal arrows at $Re=80$ across the stability boundary indicate the range of the alternating magnetic field discussed in Fig. 5. The inset illustrates the bifurcation scenario of the dominant mode amplitude $|u_{0,1}|$ for static, and two different modulated driven magnetic fields at parameters as indicated ($\Omega _H=100$) at $Re=80$. (**b**) Surface illustrating the critical Reynolds number $Re_c$ over $(s_{z,S},s_{z,M})$-plane. (**c**) Cross sections of (**b**) for different static field strength $s_{z,S}$ as indicated. The location of the bifurcation thresholds moves towards larger critical Reynolds number $Re_c[s_z(t)]$ (in high frequency limit). The inset shows the relative distance $\epsilon$ from the respective onset for static fields versus the squared modulation amplitudes $(s_{z,M})^2$.

Such static fields have been studied in detail in numerous works^22–25^ with the common result that any applied magnetic field regardless it’s orientation *stabilizes* the CCF basic state. Thus the bifurcation thresholds for primary instability (of TVF) are moved to larger *Re* with increasing field strength $s_{z,S}$ (Fig. 2a). Without magnetic fields, i.e. $s_z=0$ the critical value is $Re^0_{c}=68.8$. Worth mentioning that other axial wavenumber (here $k=3.927$) will lead to other critical Reynolds numbers $Re^0_{c}$. The stabilization can be approximated with a power law according to $Re_{c}(s_{z,S}) = Re^0_{c} + a_1 s_{z,S}^2$ (with $a_1=53.3$) (Fig. 2a).

#### Modulated magnetic fields ($s_{z,M}\ne 0$)

Similar to increasing field strength $s_{z,S}$ for static magnetic fields, also an increase in the modulation amplitude $s_{z,M}$ stabilizes the system. Figure 2b presents the surface of $Re_c$ (over $(s_{z,S},s_{z,M})$-plane) which is convex in all points in any direction. Thereby the quantity of stabilization also increases with increasing modulation amplitude (Fig. 2c). Although this behavior remains qualitative the same for alternating magnetic fields with different static contributions $s_{z,S}$, the relative effect weakens with increasing the static contribution. For parameters in Fig. 2 the maximum stability enhancement in *Re* is about $220\%$, comparing the system in absence of any magnetic field with alternating magnetic field at $(s_{z,S}=1=s_{z,M})$. The inset in Fig. 2c presents the variation of reduced value $\epsilon =Re_c[s_z(t)]/Re^0_c(s_{z,S})-1$ against the squared modulation amplitudes $(s_{z,M})^2$. The stabilization of the CCF basic state can be quantified with an approximate power law according to $Re_{c}[s(t)] = Re^0_{c}(s_{z,S}) + a_1 s_{z,S}^2$ (with $a_1=29.5$), where $Re^0_{c}(s_{z,S})$ is the stability threshold in presence of a static magnetic field. The decreasing slopes $\Delta \epsilon / \Delta (s_{z,M})^2$ (inset in Fig. 2c) originate from the stronger stabilization effect in pure static magnetic fields.

In terms of stability one can summarize, that the system reacts to an alternating modulation of the magnetic field similar as increasing the magnetic field strength in the static case. The stronger stabilization with increasing modulation amplitude originates from the static field behavior in particular it’s non-linear grows with power of 2 (Fig. 2a). Thus during one modulation period the system experience a stronger stabilization effect while the modulation amplitude is above the average field strength in comparison to the de-stabilization in the other half period. As result (for high frequency) the stabilization within an alternating magnetic field corresponds to a static field strength, which lies *above* the mean value of the alternating field. For the same reason the stabilization also grows with increasing modulation amplitude (Fig. 2c).

#### Bifurcation behavior

**Figure 3:**
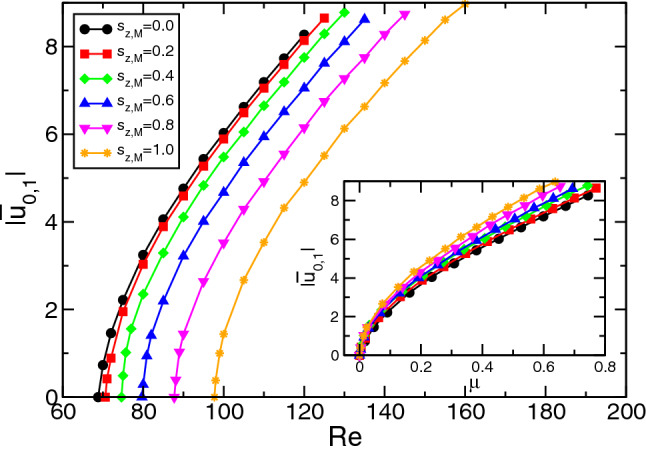
Mode amplitudes $|u_{0,1}|$ of (dominant) radial flow field amplitudes of TVF at mid gap versus Reynolds number *Re*. The inset shows the same but using the relative distance $\mu = Re(s_{z,S})/Re^0_c-1$, of the Reynolds number *Re* from the respective onset with different modulation amplitudes $s_{z,M}$ in the magnetic fields as indicated.

Figure 3 illustrates the stable forward bifurcating branches of TVF solutions for different modulation amplitudes $s_{z,M}$ as indicated ($s_{z,S}=0$) of the magnetic field. The onsets corresponds to the critical curve in absence of any magnetic field $s_{z,S}=0$ (Fig. 2 in main paper). Being supercritical the dominant mode amplitudes $|u_{0,1}|$ grow in well known square root manner. For better comparison we also consider use the relative distance $\mu = Re(s_{z,S})/Re^0_c-1$ (inset), with $Re^0_{c}$ (depending on system parameters, e.g. the axial wavenumber *k*) being the Reynolds number for which the flow becomes supercritical in the absence of a magnetic fields.

As a result one can say, increasing the modulation amplitude $s_{z,M}$ moves the onset of instability to larger control parameters (*Re*, to the right in Fig. 3) and therefore stabilizes the CCF basic state. In addition it also slightly effects/modifies the bifurcation characteristics itself. Rescaling the bifurcation scenario by the corresponding onsets (insets in Fig. 3) one sees that with increasing modulation amplitudes $s_{z,M}$ also the mode amplitudes grow faster, the corresponding slopes become steeper. Similar observation numerical and experimental has been already found for increasing field strength in static magnetic fields^22–24^.

A further bifurcation scenario of the dominant mode amplitudes $|u_{0,1}|$ for static non-zero, and two different modulated driven magnetic fields (also with finite static contribution) is shown at the inset in Fig. 2a ($\Omega _H=100, Re=80$). The chosen parameters (cf. horizontal arrows in Figs. 1b, 2a) correspond to those for which in the following the dynamic system response will be investigated. For larger modulation amplitude $s_{z,M}=0.2$, TVF dissapear at smaller corresponding static control parameter $s_{z,S}$ and the system returns to the CCF basic state. This is another confirmation for stabilization effect with increasing modulation amplitude $s_{z,M}$.

### Dynamic system response

#### Supercritical flow state

**Figure 4:**
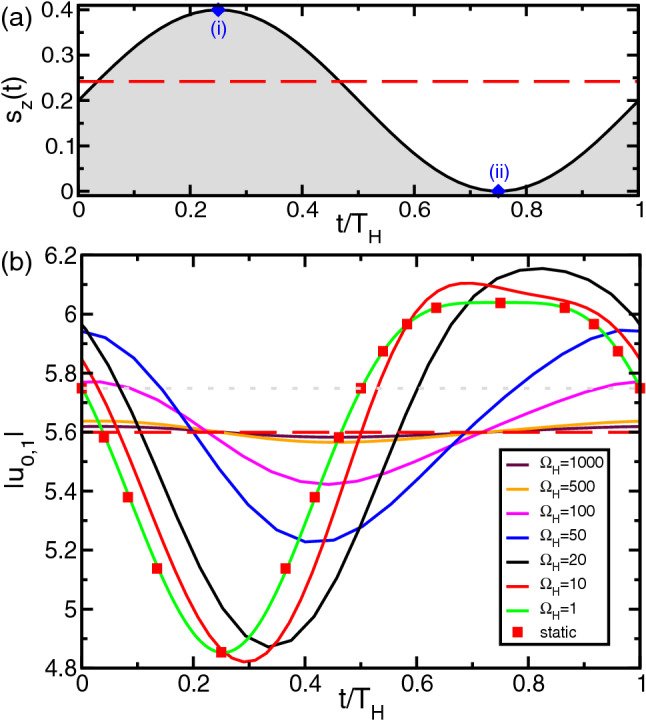
Supercritical TVF in magnetic fields with different driving frequencies $\Omega _H$. (**a**) Temporal oscillations of the control function $s_z(t) = s_{z,S} + s_{z,M}\sin {(\Omega _h t)}$ ($s_{z,S}=0.2, s_{z,M}=0.2$). (**b**) The dominant mode amplitude $|u_{0,1}|$ as a function of the reduced time $t/T_H$ ($T_H=2\pi /\Omega _H$ being the modulation period associated with the corresponding frequency). The red squares show the stationary response to stationary magnetic field with magnetic field strength $s_z$ given by the actual value of $s_z(t)$. The dashed red lines show the order parameter for stationary driving with the mean Niklas parameter $\langle s_z(t)\rangle =0.245$. Further control parameter $Re=100$.

Consider supercritical TVF at $Re=100 \Leftrightarrow \mu =Re(s_z(t))/Re^0_c=0.453$ (far away from the onset of instability $Re_c(s_z=0)=68.8$). Figure 4 shows the oscillation of the control function $s_z(t)$ together with the system response, illustrated by the mode amplitudes $|u_{0,1}|$ as a function of the reduced time $t/T_H$ ($T_H=2\pi /\Omega _H$ being the associated modulation period). The temporal oscillations are shown for different frequencies $\Omega _H$ as indicated.

In the high-frequency limit, solely the time average of $s_z(t)$ affects the stability behavior. Thus, in this limit the stability boundary coincides with a static stability boundary using an equivalent static magnetic Niklas parameter. Note, that this *larger* than the mean value $\langle s_z(t)\rangle _{T_H}$ (cf. Fig. 4a). For the given field the order parameters for equivalent static driving is $s_z=0.245$, which, for the sake of reference is also included (red dashed lines) in Fig. 4 (Note here $\langle s_z(t)\rangle _{T_H}=0.2$). For the modulation with the high frequency $\Omega _H\gtrsim 100$, the flow dynamics is nearly averaged. Variations in the dominant mode amplitude $|u_{0,1}|$ are small compared to its mean value. For $\Omega _H=100$ the modulation amplitude $\Delta |u_{0,1}|$ is barely $0.29\%$ of its time mean (Fig. 4b). A phase shift between the maximum and minimum of field function $s_z(t)$ versus the minimum and maximum of the mode amplitudes $|u_{0,1}|$ occurs: the latter ones are temporally delayed to the former because of the inertia of the fluid resisting the fast changing accelerating Kelvin force leading to this time lag. Consistently the phase shift decreases with decreasing frequency (best visible for $|u_{0,1}|$ in Fig. 4b). Thereby the oscillation amplitudes are increasing with smaller $\Omega _H$. The lower the modulation frequency, the closer the oscillation profiles get to the curve of a static magnetic field (red squares). Deviations just persist in the vicinity of the bifurcation threshold, because the dynamics become infinitely slow there.

The inharmonic behavior in the mode amplitudes $|u_{0,1}|$ (Fig. 4b) for very low frequency (and the static limit) reflects the increasing effect onto the flow dynamics with increasing field strength $s_z(t)$ (Fig. 2). For $\langle s_z(t)\rangle =0.2$ the system is supercritical with $\mu \approx 0.4 (Re_c=77.7)$ which over one period decreases to (i) $\mu =0.29 (Re_c=71.3)$ (at max$(s_{z,M})=0.2$) and increases to (ii) $\mu \approx 0.45 (Re_c=68.8)$ (at min$(s_{z,M})=0.0$). Thus, the stabilization effect is significant stronger for positive modulation amplitude. Figure 4b reflects this by either steeper/larger variation $\Delta |u_{0,1}|$ for positive modulation amplitude $s_{z,M}>0$ as well as a much flatter profile $|u_{0,1}|$ for negative modulation amplitude $s_{z,M}<0$. The latter is a direct consequence of smaller variation in $Re_c$ with small field strengths (Fig. 2a). Worth mentioning, although not further studied such inharmonic response behavior has been earlier reported in Rayleigh–Bénard system exposed to a time-periodic magnetic field^34^.

Interesting observation is the fact that for low frequencies $\Omega _H$, approaching the static state the mode amplitudes $|u_{0,1}|$ within one period slightly overshoot the maximum and minimum values of their static counterparts. For high frequencies $\Omega _H\gtrsim 30$ the mode amplitudes $|u_{0,1}|$ move around the average well within their maximum and minimum limits. It is the inertia of the fluid itself which causes this overshooting.

#### Walking the ridge of instability

In the following we will focus on an alternating magnetic field for such parameters that the system changes between subcritical and supercritical response over one period of driving. Consider for $Re=80$ an alternating magnetic field with $s_{z,S}=0.4$ and $s_{z,M}=0.1$ or $s_{z,M}=0.2$. While in the pure static case the system is supercritical (cf. point B in Figs. 1, 2), it becomes temporally subcritical for both considered modulation amplitudes.

**Figure 5:**
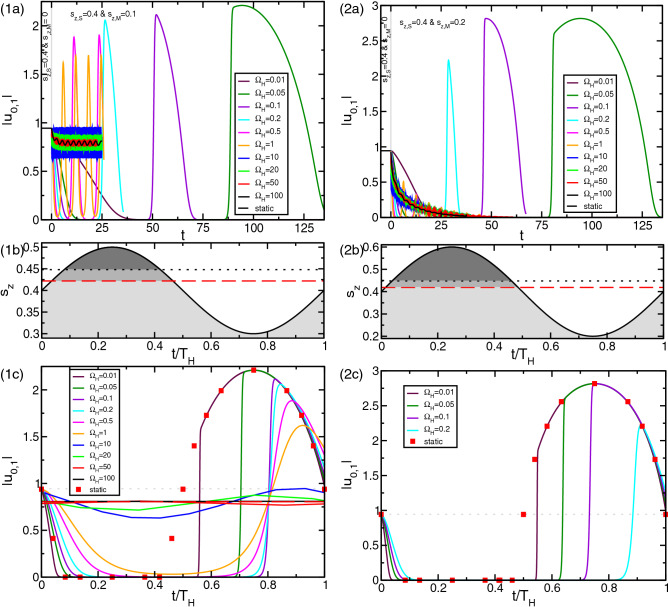
Non-linear system response around the instability. (**a**) Time evolution of the dominant mode amplitude $|u_{0,1}|$ as a function of time for different $\Omega _H$ as indicated and modulation amplitudes (1) $s_{z,M}=0.1$ and (2) $s_{z,M}=0.2$, respectively (cf. trajectory III and IV in Fig. 1). Either of these modulation starts at $t=0$, before only a static field $s_{z,S}=0.4$ ($s_{z,M}=0.0$) is present. For clarity/visibility mode amplitudes $|u_{0,1}|$ are only shown until $t=25$ in case of $\Omega _H\geqslant 0.5$ (**1a**). (**b**) Temporal oscillations of the control function $s_z(t) = s_{z,S} + s_{z,M}\sin {(\Omega _h t)}$. The dotted black and dashed red lines mark the stationary ($s_{z,S}=0.4$, $s_{z,M}=0.0$) and high frequency limit oscillatory ($s_{z,S}=0.4$, $s_{z,M}=0.1$) bifurcation threshold, respectively. (**c**) as (**a**) but as a function of the reduced time $t/T_H$. The red squares show the response to stationary magnetic field with magnetic field strength $s_z$ given by the actual value of $s_z(t)$. The red dashed line in (1*c*) indicates the (time averaged) mode amplitudes for modulated driving ($\Omega _H=50,\, 100$ almost falls on top of it). Note, that in (1) for modulated driving with $\Omega _H \gtrsim 0.27$ the system remains supercritical. Other for (2) at which for modulated driving with $\Omega _H \gtrsim 0.27$ the system remains subcritical. Further control parameter $Re=80$.

#### Small modulation amplitude ($s_{z,M}=0.1$*)*

The system becomes only slightly subcritical over one period. In the high frequencies limit the time averaged magnetic field $\langle s_z(t)\rangle$ for modulated driving (dashed red line in Fig. 51c) corresponds to a static magnetic field with $s_{z,S}\approx 0.423$ (cf. inset in Fig. 2). With decreasing frequency $\Omega _H$ first the amplitude in the oscillating mode $|u_{0,1}|$ continuously increase before at $\Omega _H\lesssim 0.5$ it eventually becomes temporally *zero* indicating that the system is now subcritical. The smaller the frequency $\Omega _H$, the longer the system remains subcritical (Fig. 51c). Over one period, for such low frequencies, a fast growth of the mode amplitude $|u_{0,1}|$ followed by a relaxing just similarly to values close to the stationary can be observed. With further decreasing $\Omega _H$ the oscillation profile in the mode amplitudes $|u_{0,1}|$ approaches the static scenario. As for full supercritical flow state (Fig. 4), a temporally delay between the extrema of $s_z(t)$ and the corresponding extrema (min and max) in the mode amplitudes $|u_{0,1}|$ appears.

#### Large modulation amplitude ($s_{z,M}=0.2$*)*

The system goes deeper into the subcritical regime within one period of driving (Fig. 5(2)) and as a result remains subcritical in the high frequency limit (see inset in Fig. 2), which is just opposite to the scenario for small modulation amplitude (1). The decay of the mode amplitudes $|u_{0,1}|$ in Fig. 5b for larger frequencies $\Omega _H$ clearly illustrates the subcriticality for here given alternating field. Corresponding equivalent static magnetic field is $s_{z,S}\approx 0.407$. However, with decreasing $\Omega _H$ the system becomes temporally supercritical. Analogous fast growth of the mode amplitude $|u_{0,1}|$ followed by a relaxing close to the stationary state appears. Again, the lower the modulation frequency $\Omega _H$, the larger the oscillation amplitudes $|u_{0,1}|$, thereby approaching the limiting value given by the respective stationary solution curve (red squares in Fig. 5(2c)).

## Discussion

We have shown that ferrofluidic Couette flow under alternating magnetic field becomes stabilized. The primary instability of TVF is moved towards larger *Re*, whereby the quantity of stabilization increases with larger modulation amplitude. This is similar to the modification due to static magnetic fields with increasing field strength. With increasing oscillation frequency the temporal evolution/response in the system decreases. The stability boundary for alternating magnetic field in high-frequency limit corresponds to a static stability boundary which is *above* the mean of the alternating magnetic field. This results from the fact that during one modulation period the system experience a stronger stabilization effect while the modulation amplitude is above the average field strength in comparison to the de-stabilization in the other half period. For very low modulation frequencies the oscillation profiles approach the stationary curves.

In addition, we found that the system response is selective to driving parameters around the primary instability. As such an alternating magnetic field can force/drive the system to be subcritical or supercritical.

**Figure 6:**
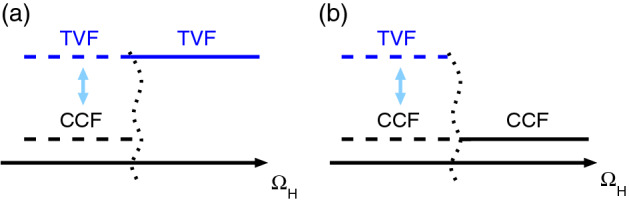
Schematic illustration for stability change with $\Omega _H$. Schematic illustration the switch between sub- and supercritical flow states with variation in the driving frequency $\Omega _H$ (increasing left to right) (cf. Fig. 5).

The schematic in Fig. 6 summarizes the non-linear system response based on small and large modulation amplitudes with respect to variation in the driving frequency $\Omega _H$. In any case the high frequency limit selects a *single* solution, the system is either sub- or supercritical. Which of the solution is selected (subcritical or supercritical) depends on the modulation amplitude. For studied parameters, $s_{z,S}=0.4$ and modulation amplitudes $s_{z,M}=0.1$ and $s_{z,M}=0.2$, respectively (Fig. 5) the selection appears at $\Omega _H \approx 0.27$. Main characteristics while “surfing the edge of instability” can be described as follows: For small magnetic modulation amplitudes the system is supercritical for high frequencies $\Omega _H$. Decreasing the frequency modifies this scenario and the system becomes temporally sub- and supercritical. On the other hand for large modulation amplitudes the system is subcritical in the high frequency limit. However, decreasing the frequency also modifies the system response to be temporally subcritical and supercritical.

The present work highlights the importance of complexer fluids under external driving. As such the variation in frequency of the alternating field provides a very *simple* and in particular accurate *controllable* way to trigger the system response to be either subcritical or supercritical. This offers various ways for industrial applications, e.g. focussing on the significant difference in torque between the subcritical CCF basic state and the primary instability of supercritical TVF.

## Methods

### Direct numerical simulation

DNS for ferrohydrodynamical flow using the Niklas approximation are employed^19,24^. In the present study, we consider in axial direction periodic boundary conditions corresponding to a fixed axial wavenumber $k = 3.927$, motivated by experimental findings for the appearance of primary TVF instability in Taylor–Couette flow with outer cylinder at rest^3,33^. No-slip boundary conditions are used on the cylinder surfaces and the radius ratio of inner and outer cylinders, is kept fixed at $r_i/r_o=0.5$. The DNS are conducted combining a standard, second-order finite-difference scheme in (*r*, *z*) with a Fourier spectral decomposition in $\theta$ and explicit time splitting. The explored parameter range spans $68\leqslant Re \leqslant 152$, $0 \leqslant (s_{z,S},s_{z,M}) \leqslant 1$, and $10^{-3}\leqslant \Omega _H\leqslant 10^3$. For these parameters, the choice of 16 azimuthal modes provides adequate accuracy. We use a uniform grid with spacing $\delta r = \delta z =0.02$ and time steps $\delta t < 1/3800$.

### Ferrohydrodynamical equation of motion

The non-dimensionalized hydrodynamical equations^25,27,35^ are given by:

2 $$\begin{aligned} (\partial _t + \mathbf{u} \cdot \nabla ) \mathbf{u} - \nabla ^2 \mathbf{u}+ \nabla p= & {} (\mathbf{M}\cdot \nabla ) \mathbf{H} + \frac{1}{2} \nabla \times (\mathbf{M}\times \mathbf{H}), \nonumber \\ \nabla \cdot \mathbf{u}= & {} 0. \end{aligned}$$

On the cylindrical surfaces, the velocity fields are given by $\mathbf{u}(r_i,\theta ,z)=(0,Re,0)$ and $\mathbf{u}(r_o,\theta ,z)=(0,0,0)$, with the the Reynolds numbers $Re=\omega _i r_i d/\nu$, where $r_i=R_i/(R_o-R_i)$ is the non-dimensionalized inner cylinder radius.

Equation (2) is to be solved together with an equation that describes the magnetization of the ferrofluid. Using the equilibrium magnetization of an unperturbed state where homogeneously magnetized ferrofluid is at rest and the mean magnetic moment is orientated in the direction of the magnetic field, we have $\mathbf{M}^{\text {eq}} = \chi \mathbf{H}$. The magnetic susceptibility $\chi$ of the ferrofluid can be approximated with the Langevin’s formula^36^, where we set the initial value of $\chi$ to be 0.9 and use a linear magnetization law. The ferrofluid studied corresponds to APG933^37^. We consider the near equilibrium approximations of Niklas^19,38^ with small $||\mathbf{M} - \mathbf{M}^\text {eq}||$ and small magnetic relaxation time $\tau$: $|\nabla \times \mathbf{u}| \tau \ll 1$. Using these approximations, one can obtain^27^ the following magnetization equation:

3 $$\begin{aligned} \mathbf{M} - \mathbf{M}^\text {eq} = c^2_N \left( \frac{1}{2} \nabla \times \mathbf{u} \times \mathbf{H} + \lambda _2 {{\mathbb {S}}} \mathbf{H} \right) , \end{aligned}$$

where

4 $$\begin{aligned} c^2_N =\tau / \left( \displaystyle 1/\chi + \displaystyle \tau \mu _0 H^2 / 6\mu \Phi \right) \end{aligned}$$

is the Niklas coefficient^19^, $\mu$ is the dynamic viscosity, $\Phi$ is the volume fraction of the magnetic material, ${{\mathbb {S}}}$ is the symmetric component of the velocity gradient tensor^27,35^, and $\lambda _2$ is the material-dependent transport coefficient^35^, which we choose to be $\lambda _2 = 4/5$^24,35,39^ based on experimental observation. Using Eq. (3), we can eliminate the magnetization from Eq. (2) to obtain the following ferro-hydrodynamical equations of motion^25,27,35^:

5 $$\begin{aligned} (\partial _{t} + \mathbf{u}\cdot \nabla ) \mathbf{u} - \nabla ^{2}{} \mathbf{u} + \nabla p_M = - \frac{ s^2_z}{2} \left[ \mathbf{H} \nabla \cdot \left( \mathbf{F} + \frac{4}{5} {{\mathbb {S}}} \mathbf{H}\right) + \mathbf{H} \times \nabla \times \left( \mathbf{F} + \frac{4}{5} {{\mathbb {S}}} \mathbf{H}\right) \right] , \end{aligned}$$

where $\mathbf{F} = (\nabla \times \mathbf{u}/2) \times \mathbf{H}$, $p_M$ is the dynamic pressure incorporating all magnetic terms that can be expressed as gradients, and $s_z$ is the Niklas parameter (Eq. (7)). Note, while in earlier studies considering static magnetic field this is a real parameter, in the present work devoted to alternating magnetic fields this is basically a time-dependent function, which we will refer to as Niklas function. To the leading order, the internal magnetic field in the ferrofluid can be approximated as the externally imposed field^25^, which is reasonable for obtaining dynamical solutions of the magnetically driven fluid motion. Equation (5) can then be simplified as

6 $$\begin{aligned}&(\partial _t + \mathbf{u} \cdot \nabla ) \mathbf{u} - \nabla ^{2} \mathbf{u} + \nabla p_M \nonumber \\&\quad = s_z^2 \left\{ \nabla ^2 \mathbf{u} - \frac{4}{5} \left[ \nabla \cdot ({{\mathbb {S}}} \mathbf{H}) \right] - \mathbf{H} \times \left[ \frac{1}{2} \nabla \times (\nabla \times \mathbf{u} \times \mathbf{H}) - \mathbf{H} \times (\nabla ^2 \mathbf{u}) \right. \left. + \frac{4}{5} \nabla \times ({{\mathbb {S}}} \mathbf{H}) \right] \right\} . \end{aligned}$$

This way, the effect of the magnetic field (here homogeneous but alternating with $\mathbf{H}_z = [H_S + H_M \sin {(\Omega _H t)}] \mathbf{e}_z$) and the magnetic properties of the ferrofluid on the velocity field can be characterized by a single parameter, the magnetic field or the (here time dependent) Niklas parameter^19^:

7 $$\begin{aligned} s_z(t)= & {} \sqrt{c_N} H_z = \sqrt{c_N} [H_S + H_M \sin {(\Omega _H t)}]\\= & \,s_{z,S} + s_{z,M} \sin {(\Omega _H t)},\nonumber \end{aligned}$$

with the two time-independent control parameters

8 $$\begin{aligned} s_{z,S} = \sqrt{c_N} H_S \quad \text {and} \quad s_{z,M} = \sqrt{c_N} H_M \end{aligned}$$

standing for the static contribution ($s_{z,S}$) and the modulation amplitude ($s_{z,M}$) of the driving, respectively.

### Numerics

The ferrohydrodynamical equations of motion Eq. (6) can be solved^24,25,27^ by combining a standard, second-order finite-difference scheme in (*r*, *z*) with a Fourier spectral decomposition in $\theta$ and (explicit) time splitting. The variables can be expressed as

9 $$\begin{aligned} f(r,\theta ,z,t)=\sum _{m=-m_{\max }}^{m_{\max }} f_m(r,z,t)\,e^{im\theta }, \end{aligned}$$

where *f* denotes one of the variables $\{u,v,w,p\}$. For the parameter regimes considered, the choice $m_{\max }=16$ provides adequate accuracy. We use a uniform grid with spacing $\delta r = \delta z =0.02$ and time steps $\delta t < 1/3800$. The system of coupled equations for the amplitudes $f_m(r,z,t)$ of the azimuthal normal modes $-m_{\max }\leqslant m \leqslant m_{\max }$ is solved with the FTCS (Forward Time, Centered Space) algorithm^40^. Further pressure and velocity fields are iteratively adjusted to each other with the method of “artificial compressibility”^41^.

10 $$\begin{aligned} dp^{(n)}= & {} -\beta {\varvec{\nabla }} \cdot \mathbf{u}^{(n)} \quad (0&\beta &1) \nonumber \\ p^{(n+1)}= &  \,p^{(n)}+dp^{(n)} \nonumber \\ \mathbf{u}^{(n+1)}= & \,\mathbf{u}^{(n)} - \Delta t {\varvec{\nabla }} (dp^{(n)}). \end{aligned}$$

The pressure correction $dp^{(n)}$ in the *n*th iteration step being proportional to the divergence of $\mathbf{u}^{(n)}$ is used to adapt the velocity field $\mathbf{u}^{(n+1)}$. The iteration loop (Eq. (10)) is executed for each azimuthal Fourier mode separately. It is iterated until ${\varvec{\nabla }}\cdot \mathbf{u}$ has become sufficiently small for each *m* mode considered—the magnitude of the total divergence never exceeded 0.02 and typically it was much smaller. Time steps were always well below the von Neumann stability criterion and by more than a factor of 3 below the Courant–Friederichs–Lewy criterion. Hereafter the next FTCS time step is executed.

For diagnostic purposes, we also evaluate the complex mode amplitudes $f_{m,n}(r,t)$ obtained from a Fourier decomposition in the axial direction:

11 $$\begin{aligned} f_m(r,z,t) = \sum _n f_{m,n}(r,t)e^{inkz}, \end{aligned}$$

where $k = 2\pi d/\lambda$ is the axial wavenumber.
